# Environmental and Lifestyle Factors Associated with Perceived Facial Age in Chinese Women

**DOI:** 10.1371/journal.pone.0015270

**Published:** 2010-12-13

**Authors:** Andrew E. Mayes, Peter G. Murray, David A. Gunn, Cyrena C. Tomlin, Sharon D. Catt, Yi B. Wen, Li P. Zhou, Hong Q. Wang, Michael Catt, Stewart P. Granger

**Affiliations:** 1 Unilever Discover, Colworth Science Park, Sharnbrook, United Kingdom; 2 Unilever Discover, Shanghai, China; The University of Queensland, Australia

## Abstract

Perceived facial age has been proposed as a biomarker of ageing with ‘looking young for one’s age' linked to physical and cognitive functioning and to increased survival for Caucasians. We have investigated the environmental and lifestyle factors associated with perceived facial ageing in Chinese women. Facial photographs were collected from 250 Chinese women, aged 25–70 years in Shanghai, China. Perceived facial age was determined and related to chronological age for each participant. Lifestyle and health information was collected by questionnaire. Bivariate analyses (controlling for chronological age) identified and quantified lifestyle variables associated with perceived facial age. Independent predictors of perceived age were identified by multivariate modelling. Factors which significantly associated with looking younger for one's chronological age included greater years of education (p<0.001), fewer household members (p = 0.027), menopausal status (p = 0.020), frequency of visiting one's doctor (p = 0.013), working indoors (p<0.001), spending less time in the sun (p = 0.015), moderate levels of physical activity (p = 0.004), higher frequency of teeth cleaning (p<0.001) and more frequent use of facial care products: cleanser (p<0.001); moisturiser (p = 0.016) or night cream (p = 0.016). Overall, 36.5% of the variation in the difference between perceived and chronological age could be explained by a combination of chronological age and 6 independent lifestyle variables. We have thus identified and quantified a number of factors associated with younger appearance in Chinese women. Presentation of these factors in the context of facial appearance could provide significant motivation for the adoption of a range of healthy behaviours at the level of both individuals and populations.

## Introduction

As society continues to be driven by image and in particular the quest for youthful appearance, the age an individual looks is a major motivating factor. In Caucasian populations perceived facial age has been proposed as a biomarker of ageing [1], [2] and ‘looking old for one’s age' has been linked to increased mortality [1], [3]. The significance of facial appearance therefore goes beyond the realms of vanity and aesthetics and becomes relevant for health and general well-being.

Previous studies which have assessed perceived age have focussed on which individual features of facial skin are associated with looking younger or older. Such reports have identified variation in skin wrinkling, evenness of colour and surface topography as being relevant to the perception of age [4]–[9]. Indeed within the current data set we have previously demonstrated the associations between perceived facial age and measures of skin wrinkling and hyperpigmentation [4]. It is clear however that other non skin-specific, facial features are also important in the perception of facial age e.g. sagging/jawline, lip structure and fullness, physiological features of the eye region and facial fat distribution [5], [7]–[9]. Perceived age therefore represents an integrated measure of not only facial skin parameters but also of the underlying physiology.

Investigations into the drivers and causes of differences in ageing appearance have identified both intrinsic and extrinsic factors as being important. These include genetic factors [10], sun exposure [11]–[13], smoking [9], [14], [15], dietary nutrients [16], body mass index [9], [17], and menopausal status [9], [18]. Each of these studies have however described the influence of such factors on either single features of facial skin e.g. skin wrinkling or on a composite model derived from a number of individual features [9]. By contrast, Christensen and colleagues have studied the influence of genetics on perceived facial age [1], [3], [5], whilst the association of circulating estrogen levels with perceived age in peri-menopausal women has also been investigated [19]. To date, only Rexbye et al, have described the influence of extrinsic/environmental factors on perceived age: low BMI, low social class, high depression symptomology and suffering from cardio-vascular disease were associated with looking older; being married or having a higher BMI were associated with more youthful appearance [20]. Whilst such studies have informed on the identity of which environmental and lifestyle factors influence perceived age in Caucasians these have been in specific and reasonably narrow population groups e.g. in peri-menopausal women [19] or those aged 70 years or older [20].

Whilst the effects of sun exposure on the appearance of Chinese skin has been described [13], [21], [22] to date there are no reports of which other extrinsic factors could be influencing skin ageing in Chinese women or more importantly of studies focussed on perceived age in this population group. We have therefore investigated the associations between perceived facial age and measures of environment, lifestyle and health in Chinese women across an age range from 25–70 years.

## Methods

A cross-sectional study was performed sampling 250 women, aged 25–70 years living in Shanghai, China. Between 4–6 women were included for each year of chronological age. Participants were in good general health with no active skin conditions; were not pregnant; had no cuts, lacerations, bruises etc on the face or neck and had not undergone any surgery or laser treatments on the face or neck (including Botox). Participants were required to provide proof of age.

The method used to determine the perceived facial age of the subjects has been described and validated previously [2], [4], [5]. Briefly, high definition digital images of the face (front view and left side view) were presented to a panel of visual assessors (with no previous age assessment experience or dermatology training). Images of subjects with their eyes closed and adopting a neutral facial expression were collected; no make up or jewellery was worn; hair was held away from the face with a hair band and the subjects' clothing was masked with a neutral coloured shawl. The photographs were divided into 14 studies each of 18 photos (7 of the studies were of younger subjects (roughly 20 to 50 years of age) and 7 of older photos (roughly 40 to 75)). Images were presented to the assessors in random order within a study and studies were done in random order. Each image was assessed by at least 75 Chinese scorers (recruited from the local area) producing a Kendall's coefficient of concordance value of 0.826 (p<0.0001). The mean standard error for an image was 0.79 years (range 0.61–0.82 years).

Participants completed general lifestyle questionnaires collecting data on socio-economic status, level of education, demographics, diet, past and present sun exposure, general health, dental health, current medications, menopausal status, allergies, smoking history, levels of physical activity and skin care product use.

All subjects provided written informed consent for the use of their data and images. Ethical approval was granted by the Unilever R&D (Shanghai) Ethics Committee, Shanghai, China.

Analysis of the lifestyle variables was carried out by using single comparisons against the difference between perceived age and chronological age (also termed age difference LSMean). For individuals who were judged to look younger than their chronological age this figure had a negative value; for subjects judged to look older than their chronological age this value had a positive value. Chronological age was included as a covariate in the Analysis of Variance (ANOVA) models performed using Proc GLM in SAS.

To investigate which of the main findings were independent of one another, a multiple regression approach was used. The difference between perceived age and chronological age was used as the primary outcome variable and only subjects with complete data sets were included in the analysis (n = 220). Only questions with more than 5 responses in at least two categories were included in the analyses. The statistical method LASSO (least absolute shrinkage and selection operator) was used to pre-select questions that potentially best modelled/explained the data (with chronological age included as a covariate). Those questions were then used in an ANOVA model. Variables were eliminated from the model using a step-wise procedure, removing at each stage the variable which was least significant in the model. To establish the least significant variable, first, for each variable, we obtained the minimum p-value over all the pair-wise comparison between levels of that variable. The least significant variable was defined to be the variable with the highest such minimum p-value. This process was repeated until the model contained only statistically significant variables. Chronological age was included as a covariate in the final model.

## Results

### Relationships between lifestyle variables and perceived facial age- bivariate analyses

Perceived age was determined for 239 women in the study, and the difference between perceived age and chronological age was related to the individual responses supplied in the lifestyle questionnaires. Those variables with a statistically significant relationship at the 95% confidence level were identified and the size of the effect in years of perceived facial age was quantified (Table 1).

**Table 1:** Bivariate analysis of lifestyle variables.

	*F-test			Age difference		Greatest difference	$F-test
Variable	p-value	Response categories	n	LSMean (st err)		between categories	p-value
Years of education	<0.001	10 years or more	139	−0.65 (0.40)	a	5.54 years	<0.001
		7–9 years	57	0.92 (0.60)	a		
		0–6 years	24	4.89 (1.02)			
Number of household members	0.027	Less than 3	39	−0.10 (0.77)	a,b	2.32 years	0.079
		3	143	−0.01 (0.39)	a		
		More than 3	38	2.22 (0.76)	b		
Menopausal status	0.020	Pre-menopausal	130	−1.04 (0.65)	a	3.51 years	0.020
		Peri-menopausal	14	−0.31 (1.31)	a,b		
		Post-menopausal	95	2.47 (0.81)	b		
Number of visits to doctor in last 12 months	0.013	7 or more	35	−0.73 (0.92)	a	4.70 years	0.007
		4–6	65	0.47 (0.59)	a,b		
		1–3	97	0.08 (0.50)	a		
		None	15	3.93 (1.28)	b		
Occupational sun exposure	<0.001	Work/worked inside	164	−0.38 (0.36)	a	6.48 years	<0.001
		Equal time inside and outside	53	0.46 (0.64)	a		
		Work/worked outside	22	6.10 (1.00)			
Current sun exposure	0.015	Not much time in sun	116	−0.30 (0.45)	a	2.86 years	0.011
		Some time in the sun	89	0.59 (0.52)	a,b		
		A lot of time in the sun	31	2.56 (0.88)	b		
How much activity during the day‡	0.004	Fairly active for short periods	54	−0.15 (0.67)	a	2.64 years	0.020
		Active for parts of the day	135	−0.15 (0.42)	a		
		Active nearly all day	50	2.49 (0.70)			
Daily teeth cleaning§	<0.001	Twice per day	184	−0.75 (0.33)		4.98 years	<0.001
		Once per day	55	4.23 (0.61)			
Facial cleanser/soap use	<0.001	4x per week or more	123	−0.80 (0.48)	a	4.27 years	<0.001
		3x per week or less	63	0.16 (0.62)	a		
		Never	53	3.47 (0.73)			
Facial moisturiser use	0.016	4x per week or more	158	−0.30 (0.40)	a	2.38 years	0.015
		3x per week or less	24	1.03 (1.00)	a,b		
		Never	57	2.08 (0.71)	b		
Facial night cream use	0.016	4x per week or more	53	−1.42 (0.71)	a	2.44 years	0.012
		3x per week or less	25	0.26 (0.99)	a,b		
		Never	161	1.02 (0.40)	b		

For each variable the number of responses (n) and the mean difference between perceived age and chronological age are given (Age difference LSMean). Chronological age was included as a covariate in all tests. The statistical confidence for each variable is given (*F-test p-value). Those responses joined by the same letter were not found to be significantly different at p<0.05. The maximum difference between any two categories is given together with the statistical confidence that the mean perceived ages for these categories differs (^$^F-test p-value). ^‡^For the question on ‘How much activity during the day’ the option of ‘not very active all day’ was an option but was not selected by any subjects. ^§^For daily teeth cleaning options of ‘less than once a day’ and ‘more than twice a day’ were also given but not selected by any subjects.

Subjects with 0–6 years of education were judged on average to look 3.97 years older than those with 7–9 years of education (p = 0.002) and 5.54 years older than those with 10 or more years of education (p<0.001). The number of members in a subject's household was also identified as relating to perceived age with subjects with more than 3 household members looking on average 2.23 years older than those with 3 household members (p = 0.027) and 2.32 years older than those with less than 3 (p = 0.079). Subjects who reported being post-menopausal (defined as having had no menstrual cycles for one year) were judged to look 3.51 years older than those who reported being pre-menopausal (p = 0.020). On average, subjects who did not visit a doctor in the past year looked 4.66 years older than those with more than 6 visits (p = 0.007) and 3.01 years older than those with 1–3 visits (p = 0.037). Subjects who reported being active nearly all day (e.g. a physically active job, housework/gardening, walking to and around shops, etc) looked 2.64 years older than those who are active for only parts of the day (p = 0.004) or for only short periods of the day (p = 0.020).

In response to questions on dental hygiene habits, subjects who reported cleaning their teeth twice a day were judged on average to look 4.98 years younger than those who reported cleaning once a day (p<0.001). The level of sun exposure was also identified as relating to perceived facial age with subjects who reported spending a lot of time in the sun judged to look 2.86 years older than those who reported not spending much time in the sun (p = 0.011). Equally, subjects who work/worked outside looked 6.48 years older than those who work/worked inside for the majority of their working life (p<0.001) and 5.64 years older than those who have spent equal time outside and inside for the majority of their working life (p<0.001).

From a series of questions on the use of personal skin care products for the face, responses were categorised as frequent use (4 times per week or more), occasional use (3 times per week or more) or never use. On average, subjects who reported never using facial soap/cleanser looked 4.27 years older than those who frequently use it (p<0.001) and 3.31 years older than occasional users (p = 0.001). Subjects who reported frequently using facial moisturiser were judged to look 2.38 years younger than those who never use it (p = 0.016) with subjects who reported using facial night cream 4×/week or more looking 2.44 years younger than those who never use it (p = 0.012).

### Relationships between lifestyle variables and perceived facial age- multivariate analyses

Multiple linear regression modelling was used to determine which of the lifestyle variables could be used to independently predict the difference between perceived age and chronological age. The final model contained six lifestyle variables which, together with chronological age, accounted for 36.5% of the variability in the difference between perceived age and chronological age (Table 2). The majority of those variables were also identified in the bivariate analyses with frequency of daily teeth cleaning (p<0.001), occupational sun exposure (p = 0.001), use of facial cleanser/soap (p = 0.075) and number of household members (p = 0.091), all included in the final multivariate model. Variables not previously identified were number of times engaging in exercise per week (p = 0.019) and the frequency of eye cream use (p = 0.029).

**Table 2:** Multiple linear regression analysis of lifestyle variables.

	* F-test			Age difference		Greatest difference	$F-test
Variable	p-value	Response categories	n	LSMean (st err)		between categories	p-value
Number of household members	0.091	Less than 3	39	1.97 (0.80)	a	2.02 years	0.033
		3	143	2.70 (0.62)	a,b		
		More than 3	38	3.99 (0.80)	b		
Occupational sun exposure	0.001	Work/worked inside	152	1.56 (0.55)	a	3.96 years	<0.001
		Equal time inside and outside	51	1.58 (0.71)	a		
		Work/worked outside	17	5.52 (1.07)			
Number of times engaging in exercise per week	0.019	Never	94	2.40 (0.63)	a	2.20 years	0.008
		1–6 days	79	2.03 (0.73)	a		
		Everyday	47	4.23 (0.77)			
Daily teeth cleaning	<0.001	Twice per day	174	0.79 (0.58)		4.20 years	<0.001
		Once per day	46	4.99 (0.74)			
Facial cleanser/soap use	0.075	4× per week or more	118	2.57 (0.63)	a,b	1.94 years	0.024
		3× per week or less	58	2.07 (0.81)	a		
		Never	44	4.01 (0.80)	b		
Facial eye cream use	0.029	4× per week or more	31	1.41 (0.90)		2.64 years	0.010
		3× per week or less	33	4.05 (0.87)	a		
		Never	156	3.21 (0.49)	a		

Lifestyle variables included in the final model are given together with the number of responses (n) and the mean difference between perceived age and chronological age are given (Age difference LSMean). Responses are given in order of those with smallest difference first. The statistical confidence for each variable is also given (*F-test p-value). Those individual responses joined by the same letter were not found to be significantly different at p<0.05. The maximum difference between any two categories is given together with the statistical confidence that the mean perceived ages for these categories differs (^$^F-test p-value).

## Discussion

Perceived age i.e. the mean age an individual looks, has been demonstrated to be related to physical and cognitive functioning [1], to survival and mortality [3], and has thus been proposed as a clinically-relevant biomarker of biological age in Caucasian populations [1], [2]. Whilst perceived age has been related to various features of skin physiology e.g. wrinkling, eveness of colour, etc [4]–[9] it has also been demonstrated to be determined (in part) by features of the underlying physiology of the face e.g. lip volume, fat distribution etc [5], [7]–[9]. Perceived age therefore a multi-factorial measure of facial ageing rather than purely a measure of skin ageing. Indeed perceived age. Thus perceived facial age is a valid measure of the overall physiological condition of the face which has clinical utility [1]; indeed clinicians have been reported to use perceived age as a measure of patient health and well-being prior to any physical examination or diagnosis [1].

Whilst the intrinsic and extrinsic factors influencing perceived age and facial appearance have been widely studied in Caucasian cohorts [3], [9]–[11], [16], [17], this is the first study to profile lifestyle factors associated with perceived age in a Chinese population group. By using high definition digital images of the subjects' face, presented in a standardised format, and using a large number of local, naïve age assessors we have developed a robust methodology for determining perceived age [2]. This measure has been used previously in Caucasian populations to investigate the features associate with looking younger [5] and in the current population group to identify the skin parameters relating to ageing appearance in Chinese women [4]. In the current analyses, by relating the difference between the perceived and chronological ages of these women with their responses to a health and lifestyle questionnaire we have not only identified, but have also quantified, the environmental and behavioural factors that were associated with looking younger or older for ones age in this group.

**Figure 1:**
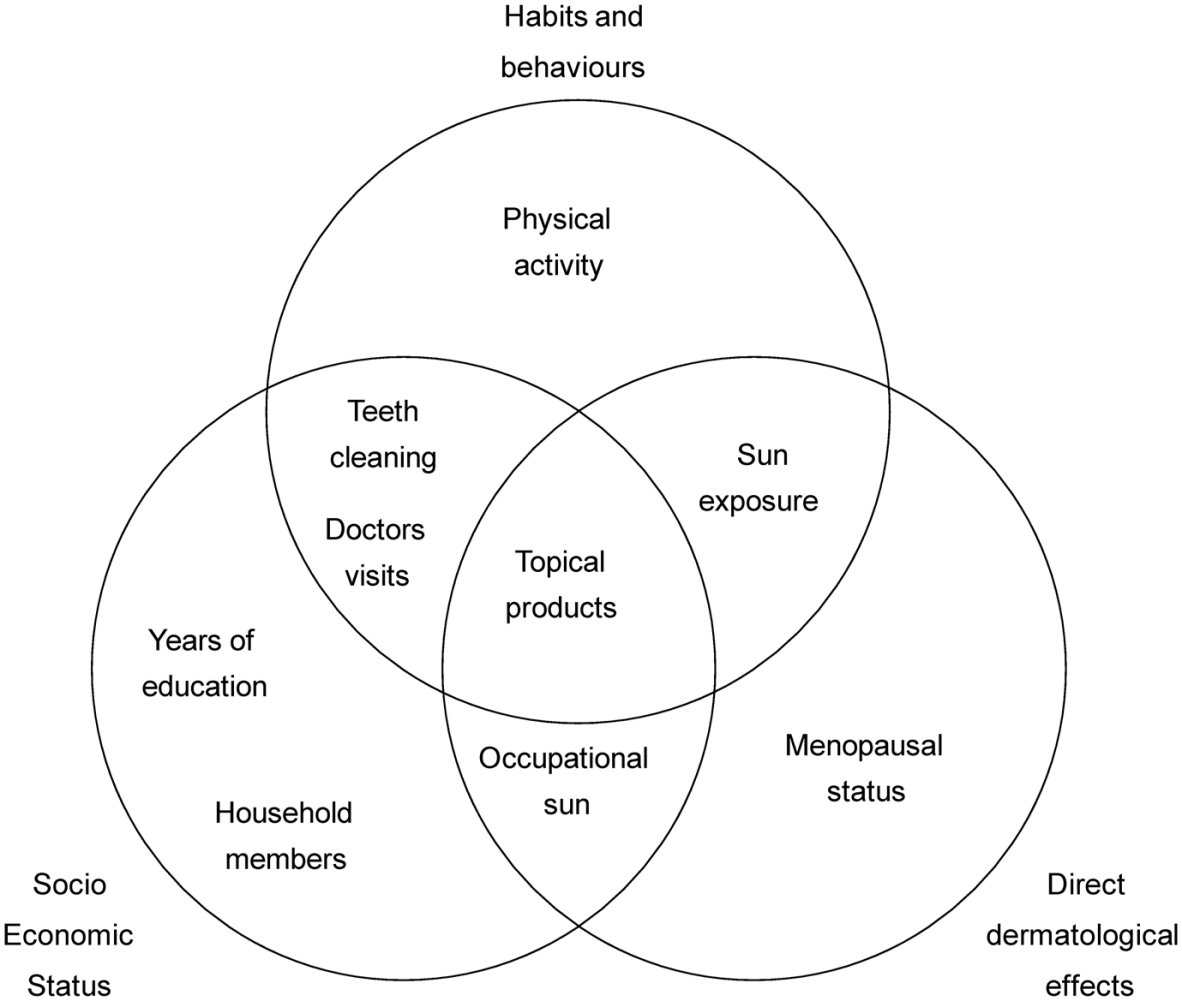
Inter-relationships of factors associated with perceived facial age. Factors were identified as statistically significant at the p<0.05 level from bivariate analyses and classified into interacting domains.

Bivariate analyses provided evidence for statistically significant associations with a number of factors which can be classified broadly into three domains relating to socio-economic status (SES), to personal habits and behaviours and to direct dermatological effects (Figure 1). This analysis re-emphasises the multi-factorial nature of the ageing process and highlights that it is the complex interplay between these factors which will ultimately influence the degree to which someone looks young or old for their age. Whilst some of the factors identified belong exclusively within a single domain (e.g. years of education within SES), others belong in two or even in all three domains. Many of these factors e.g. years spent in education, housing conditions, physical activity etc have also been identified in studies into ‘successful ageing’ in Chinese populations [23], [24]. This agreement with more established metrics of healthy ageing also provides evidence that perceived age is a valid and relevant measure in Chinese cohorts as well as in Caucasian groups [1], [3] and further reinforces the potential utility of perceived facial age as a non-invasive biomarker of health and ageing.

For some factors there are clear and established links with skin physiology and ageing appearance. For example, those women who reported being post-menopausal at the time of the study were judged on average to look 3.51 years older than those who were pre-menopausal (analysis controlled for chronological age). This is consistent with the known effects of the menopause and declining oestrogen levels on skin physiology (reviewed in Verdier-Sévrain et al [18]) and also agrees with the reported correlation between perceived age and circulating oestrogen levels in peri-menopausal women [19]. This is likely to be a solely biological effect and unlikely to be related to behavioural or socioeconomic factors.

The complex interplay between the 3 main domains is highlighted by the finding that occupational sun exposure is related to perceived age, with those who work/worked outdoors looking on average 6.48 years older than those who work/worked indoors. Whilst this factor is likely to directly influence perceived age via the well-established effects of ultraviolet light on skin i.e. photo-ageing [12], [13], [21], [22] there is also a socio-economic dimension since those women of higher SES are more likely to have jobs indoors than those of lower SES. Discrepancies in SES have been extensively reported to influence a number of health-related measures e.g. cardiovascular risk, mortality etc. [25]–[30] and the current study would propose to add perceived facial age to that list for Chinese women. That components of SES were identified in the current study is also consistent with the findings of Rexbye et al who identified low social class as associating with a more aged appearance in Northern European women [20]. The interplay of direct dermatological effects and behavioural influences on perceived age is also demonstrated by the finding that those who reported spending the greatest amount of time in the sun were judged on average to look 2.86 years older than those who spent least time.

The number of years spent in education was found to be significantly associated with perceived age and has similarly been reported to relate to ‘successful ageing’ in a previous study within a Chinese population [23]. Whilst this factor is unlikely to have a direct influence on the physiology of facial appearance, it will none-the-less have a significant bearing on household income/salaries, occupational status, access to health care, etc some of which were also identified as relating to perceived age. For example, those women who reported not visiting their doctor in the preceding 12 months were judged to look significantly older than those who went more frequently. Whilst this may be counter-intuitive in terms of an expected relationship between good health (fewer doctors visits) and younger facial appearance, given that access to medical treatment in China is funded either by the individual or by their employer, and therefore linked to SES, those visiting their doctors more regularly may be more accurately categorised as ‘health conscious with access to medical care’ rather than as ‘ill and requiring medical care’. It is a limitation of the current study that no information was gathered on the nature of the conditions for which medical care was sought or on any course of treatments administered.

Together with higher SES and direct dermatological factors which are associated with younger facial appearance, personal behaviours were also found to be important. For example, those who cleaned their teeth twice a day and those who were reasonably active during the day were found on average to look younger than those who did not. Whether these behaviours are directly influencing perceived age or are acting as proxy measures for a generally healthy lifestyle in this group would require further investigation and intervention studies. Recent epidemiological literature does however provide evidence that engaging in a series of ‘healthy behaviours’ e.g. not smoking, taking physical activity, eating fruits and vegetables can have a profound influence on a range of health parameters and even on the risk of mortality [31]–[33]. Similar studies specifically in Chinese groups have identified similar factors and reached similar conclusions [23], [24].

A cluster of variables related to frequent use of topical face care products (moisturiser, night cream, cleanser/soap) was also found to associate with younger perceived age. In addition to the direct beneficial effects of such products on skin physiology (for example see Hawkins et al, [34]), frequent use of a personal care regime also requires a behavioural commitment, with access to such products also potentially constrained by SES and relative wealth. Thus, these variables draw on all three environmental domains identified as relating to perceived facial age.

It is a limitation of the current study that it was cross-sectional in design and that self-report questionnaires were used to identify variables associated with perceived facial age. Further intervention or longitudinal studies will be required in order to demonstrate causal relationships for these factors and, where possible, these should be performed to include objective measures of the variables e.g. accelerometry to assess physical activity [35]. In addition, more in depth analyses will be needed to better understand the influences of these variables on perceived age e.g. the location, duration and intensity of optimal physical activity. Additional environmental factors which could influence perceived facial age include smoking [9], [14], [15] and dietary habits [16]. In the current population as only four women reported being current smokers no meaningful statistical test could be performed to determine an effect in this group. No information was collected on dietary habits or nutritional intakes. Whilst this study sampled 250 women equally spread over the age range 25–70 years it is envisaged that larger cohort studies will be required to confirm the current findings and to test specific hypotheses generated from the current analyses. Equally with such larger studies and sufficient subject numbers, age-group specific associations could be investigated.

In conclusion, we have identified and quantified a number of features of daily living which associated with younger perceived facial age in a group of Chinese women. Whilst many of these lifestyle factors have the potential to influence skin ageing, only a small number could reasonably be expected to relate specifically to changes in the skin with age. Many of the lifesyle factors identified in the current study have also been associated with other ‘successful ageing’ outcomes in Chinese populations supporting the belief that perceived age is a biomarker of healthy behaviours. In an increasingly image-driven world it will be interesting to assess whether facial appearance could be used as a motivating feature for the adoption of a range of healthy behaviours. We believe that in addition to younger facial appearance, individuals may also experience other personal health benefits, and at a population level, adoption of healthier behaviours may provide significant public health benefits.
